# Supplementary material for: A Practical Method for Amino Acid Analysis by LC-MS Using Precolumn Derivatization with Urea
Source: Int J Mol Sci. 2023 Apr 15;24(8):7332. doi: 10.3390/ijms24087332 (PMC10138926; doi:10.3390/ijms24087332)
Supplement: Supplementary file 1 [file ijms-24-07332-s001.zip › ijms-2270298-supplementary.pdf]

Supporting Information

A practical method for amino acid analysis by LC-MS using precolumn derivatization with urea

Runjin Zhao[a,b], Biling Huang[a, b], Gang Lu[a], Songsen Fu[a, b] *, Jianxi Ying[a, b] * and Yufen Zhao[a, b, c, d]

a Institute of Drug Discovery Technology, Ningbo University, Ningbo, 315211, P.R. China

b Qian Xuesen Collaborative Research Center of Astrochemistry and Space Life Sciences, Ningbo University, Ningbo, 315211, P.R. China

c Department of Chemical Biology, College of Chemistry and Chemical Engineering, and the Key Laboratory for Chemical Biology of Fujian Province, Xiamen University, Xiamen, 361005, P.R. China

d Key Lab of Bioorganic Phosphorus Chemistry & Chemical Biology, Department of Chemistry, Tsinghua University, Beijing, 100084, P.R. China

Corresponding author: *Jianxi Ying, yingjianxi@nbu.edu.cn

*Songsen Fu, fusongsen@nbu.edu.cn;

Contents

Figure S1. EIC-MS profile of Phe and Carbamoy-Phe. 3

Figure S2. EIC-MS profile of the product carbamoyl-Phe. 4

Figure S3. LC-MS-EIC profile of control sample and derivatization reaction sample of Ala. 5

Figure S4. LC-MS-EIC profile of control sample and derivatization reaction sample of Phe. 6

Figure S5. LC-UV profile of Ala and Carbamoy-Ala (210 nm). 7

Figure S6. LC-UV profile of Phe and Carbamoy-Phe (210 nm). 8

Figure S7. The yield of carbamoyl-Phe under different pH conditions (3-11). 9

Figure S8. EIC-MS profile of carbamoyl-Ala under different pH conditions (3-11). 10

Figure S9. EIC-MS profile of carbamoyl-Phe under different pH conditions (3-11). 11

Figure S10. The yield of carbamoyl-Phe under different temperature conditions (37 °C -80 °C). 12

Figure S11. EIC-MS profile of carbamoyl-Ala under different temperature conditions (37 °C -80 °C). 13

Figure S12. EIC-MS profile of carbamoyl-Phe under different temperature conditions (37 °C -80 °C). 14

Figure S13. The yield of carbamoyl-Phe under different time conditions (1-8 h). 15

Figure S14. EIC-MS profile of carbamoyl-Ala under different time conditions (1-8 h). 16

Figure S15. EIC-MS profile of carbamoyl-Phe under different time conditions (1-8 h). 17

Figure S16. EIC-MS profile of 20 amino acid before and after derivatization. 18

Figure S17. EIC-MS profile of 3,3'-disulfanediylbis(2-ureidopropanoic acid). 19

Figure S18. EIC-MS profile of N^2, N^6-dicarbamoyllysine. 20

Figure S19. Derivatization of complex sample. 21

Figure S20. EIC-MS profile of carbamoyl-Phe-Phe. 22

Table S1 EIC-MS extraction range of AA and CAA. **23**

Table S2 Changes in retention time before and after AA derivatization **24**

Table S3 Derivatized conversion rate of 20 AA Mixtures. **25**

Table S4. Formulation of cell culture medium DMEM. **26**

Table S5. Formulation of cell culture medium RPMI 1640. .... **27**

Table S6 Derivatized conversion rate of AAs in Cell culture medium DMEM. **28**

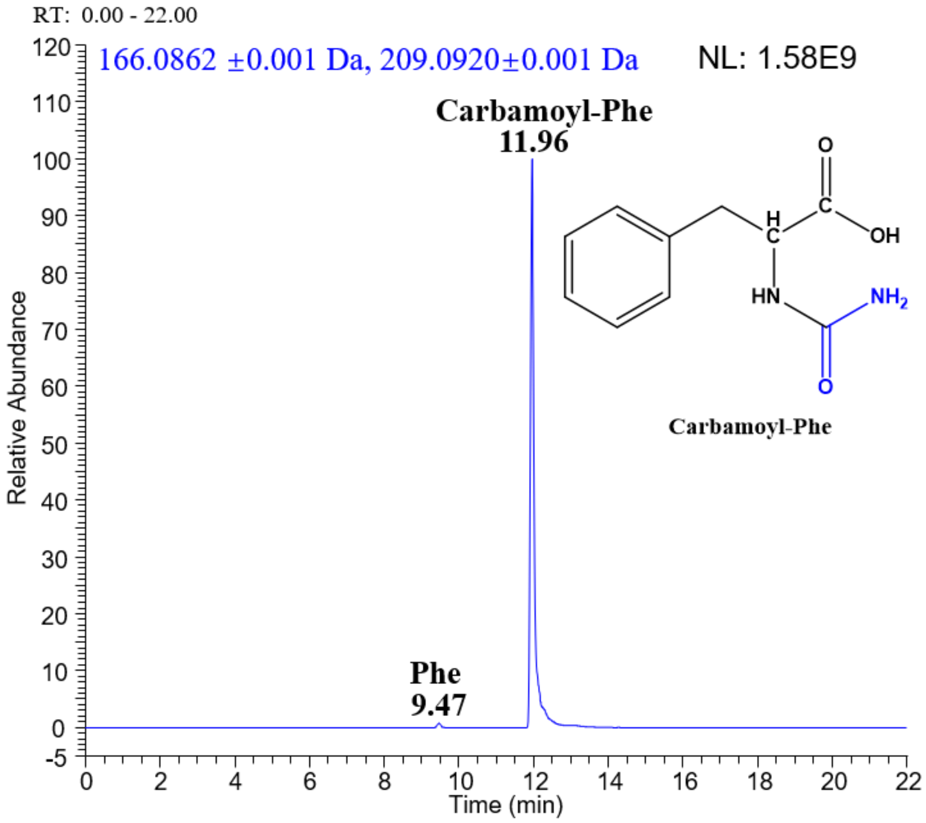

Figure S1. EIC-MS profile of Phe and Carbamoy-Phe. HPLC conditions for single amino acid derivatives was as follows: mobile phase A: deionized water (0.1% formic acid); mobile phase B: acetonitrile. Gradient program was as follows: 0-5 min, 5%; 5–13 min, 5-75%; 13-15 min, 75%; 15-17 min, 75-5%, 17-22 min, 5% B. The eluent flow rate was 1 mL /min and the column was maintained at 30°C and 8 μL of the sample was injected.

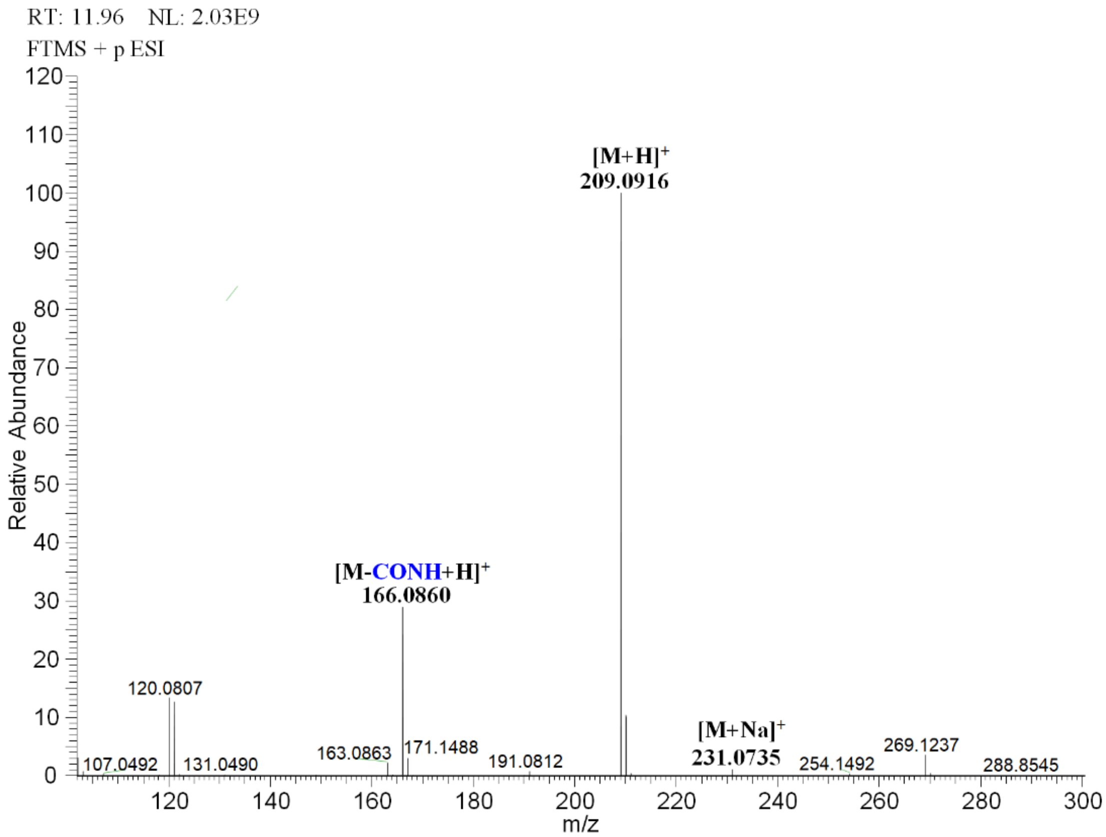

Figure S2. EIC-MS profile of the product carbamoyl-Phe.

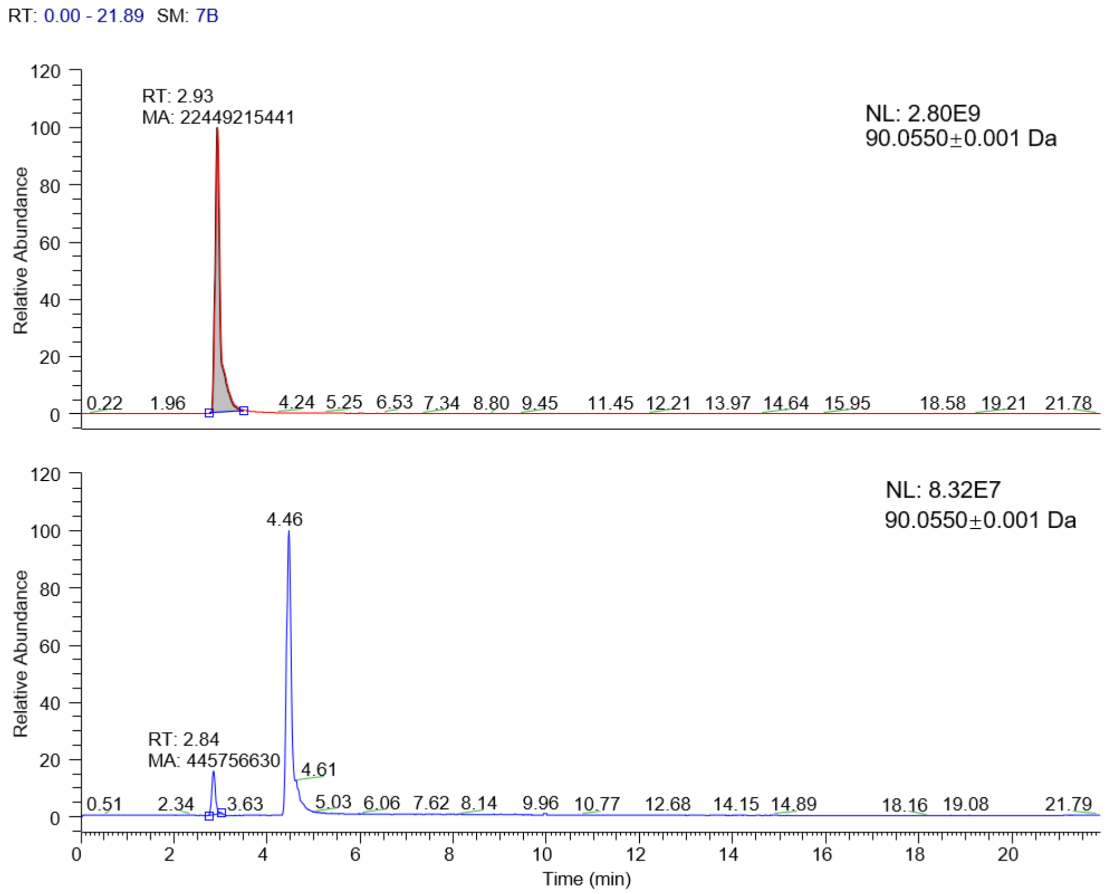

Figure S3. LC-MS-EIC profile of control sample and derivatization reaction sample of Ala. The calculated value of Ala [M+H]+ is 90.0550. The peak area of control sample (non-urea added) is 22449215441, and the peak area of residual Ala after derivatization reaction is 445756630. The conversion rate=(1-(445756630/22449215441)) × 100%=98.01%. HPLC conditions for single amino acid derivatives were as follows: mobile phase A: deionized water (0.1% formic acid); mobile phase B: acetonitrile. Gradient program was as follows: 0-5 min, 5%; 5–13 min, 5-75%; 13-15 min, 75%; 15-17min, 75-5%, 17-22 min, 5% B. The eluent flow rate was 1 mL /min and the column was maintained at 30°C and 8μL of the sample was injected.

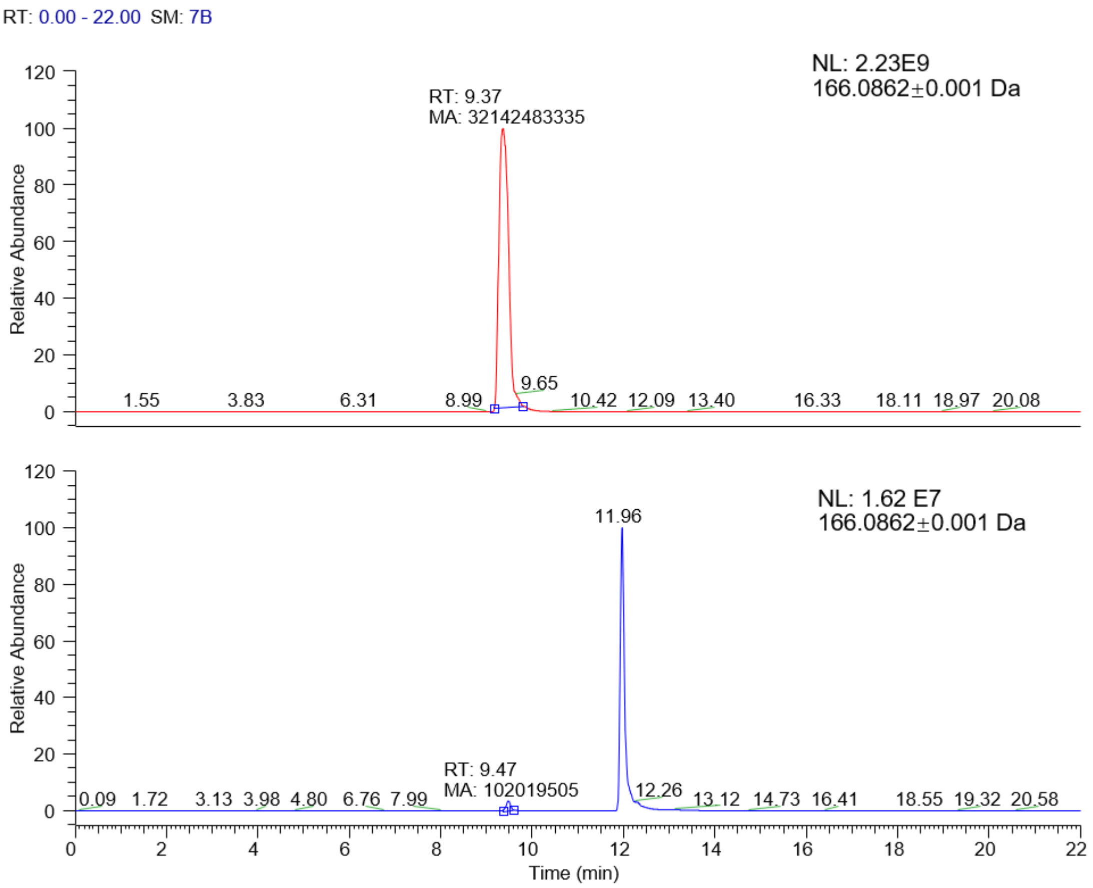

Figure S4. LC-MS-EIC profile of control sample and derivatization reaction sample of Phe. The calculated value of Phe [M+H]+ is 166.0862. The peak area of control sample (non-urea added) is 3214248335, and the peak area of residual Phe after derivatization reaction is 102019505. The conversion rate=(1-(102019505/3214248335)) × 100%=99.69%. HPLC conditions for single amino acid derivatives were as follows: mobile phase A: deionized water (0.1% formic acid); mobile phase B: acetonitrile. Gradient program was as follows: 0-5 min, 5%; 5–13 min, 5-75%; 13-15 min, 75%; 15-17min, 75-5%, 17-22 min, 5% B. The eluent flow rate was 1 mL /min and the column was maintained at 30°C and 8μL of the sample was injected.

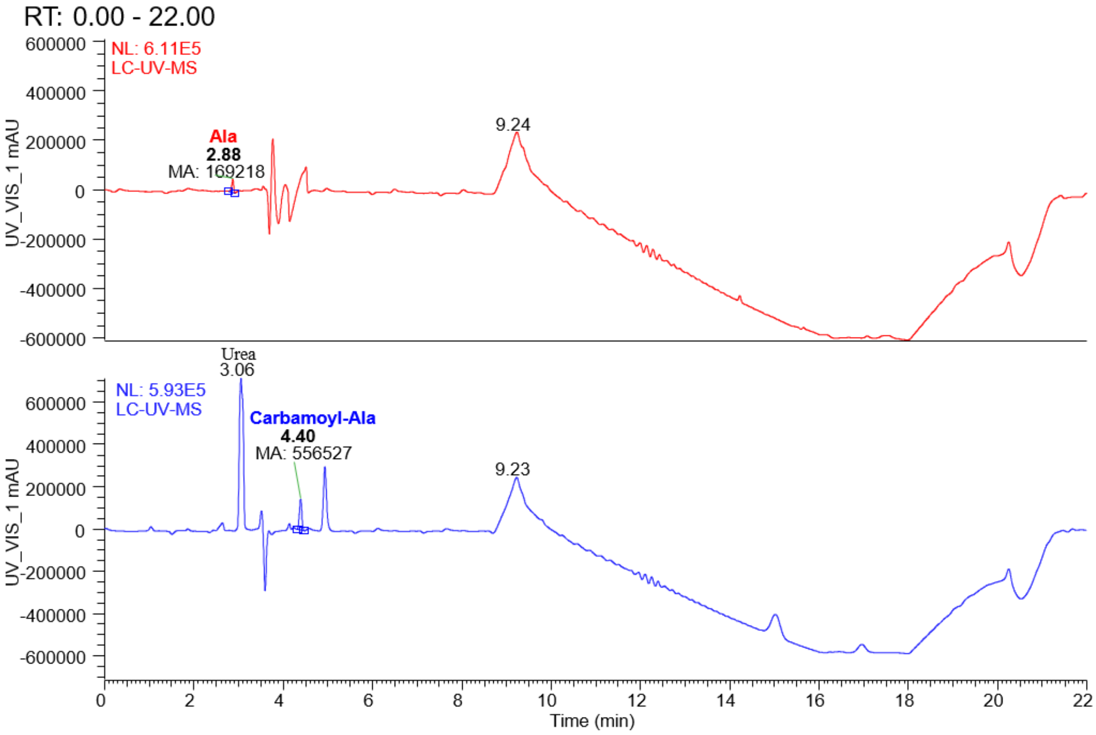

Figure S5. LC-UV profile of Ala and Carbamoyl-Ala (210 nm). NL values from the UV peaks of Ala and Carbamoyl-Ala. UV(Carbamoyl-Ala)/UV(Ala)=556527/169218=3.29. HPLC conditions for single amino acid derivatives were as follows: mobile phase A: deionized water (0.1% formic acid); mobile phase B: acetonitrile. Gradient program was as follows: 0-5 min, 5%; 5–13 min, 5-75%; 13-15 min, 75%; 15-17 min, 75-5%, 17-22 min, 5% B. The eluent flow rate was 1 mL /min and the column was maintained at 30°C and 8 μL of the sample was injected.

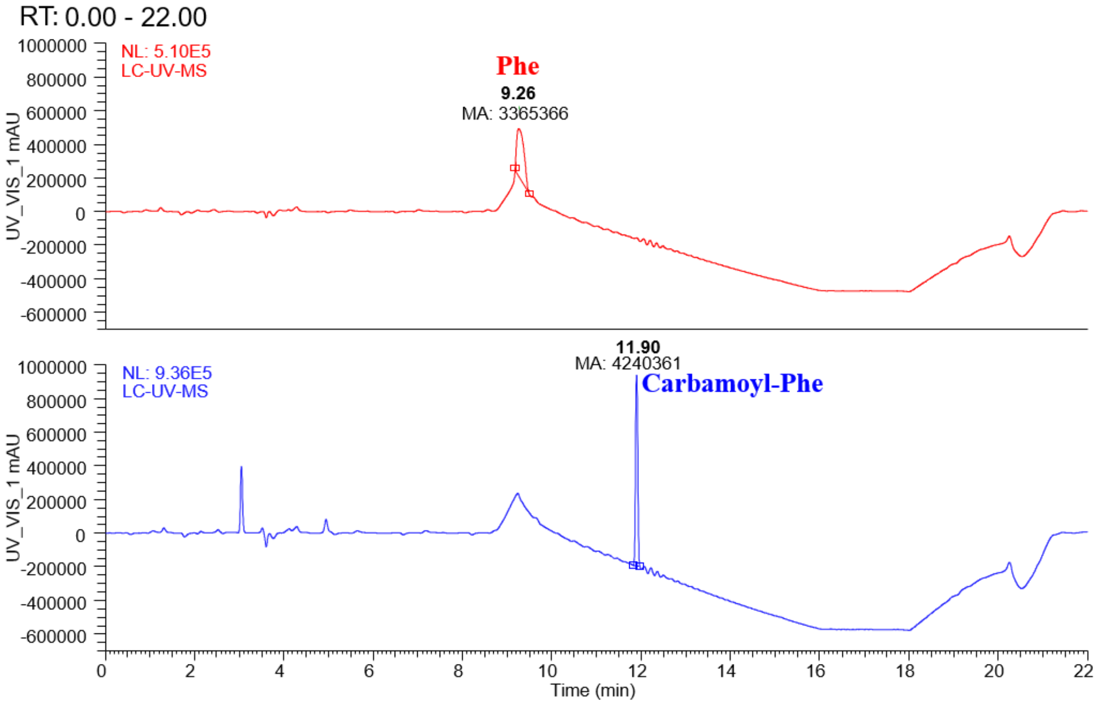

Figure S6. LC-UV profile of Phe and Carbamoyl-Phe (210 nm). NL values from the UV peaks of Phe and Carbamoyl-Phe. UV(Carbamoyl-Phe)/UV(Phe)=4240361/3365366=1.26. HPLC conditions for single amino acid derivatives were same as Figure S5.

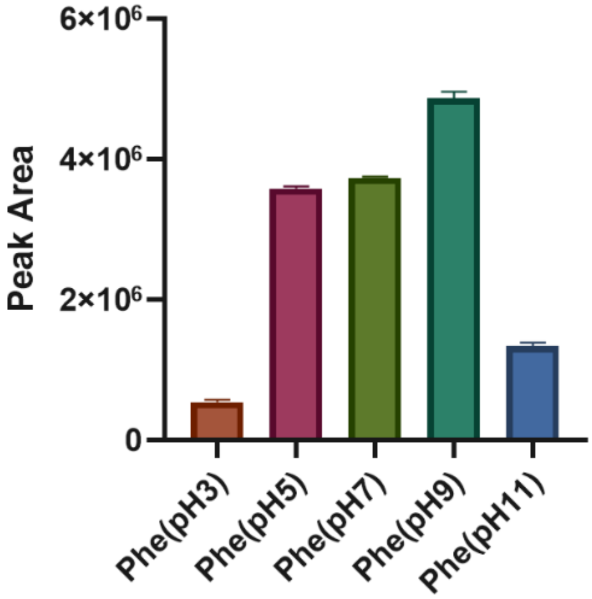

Figure S7. The yield of carbamoyl-Phe under different pH conditions (3-11).

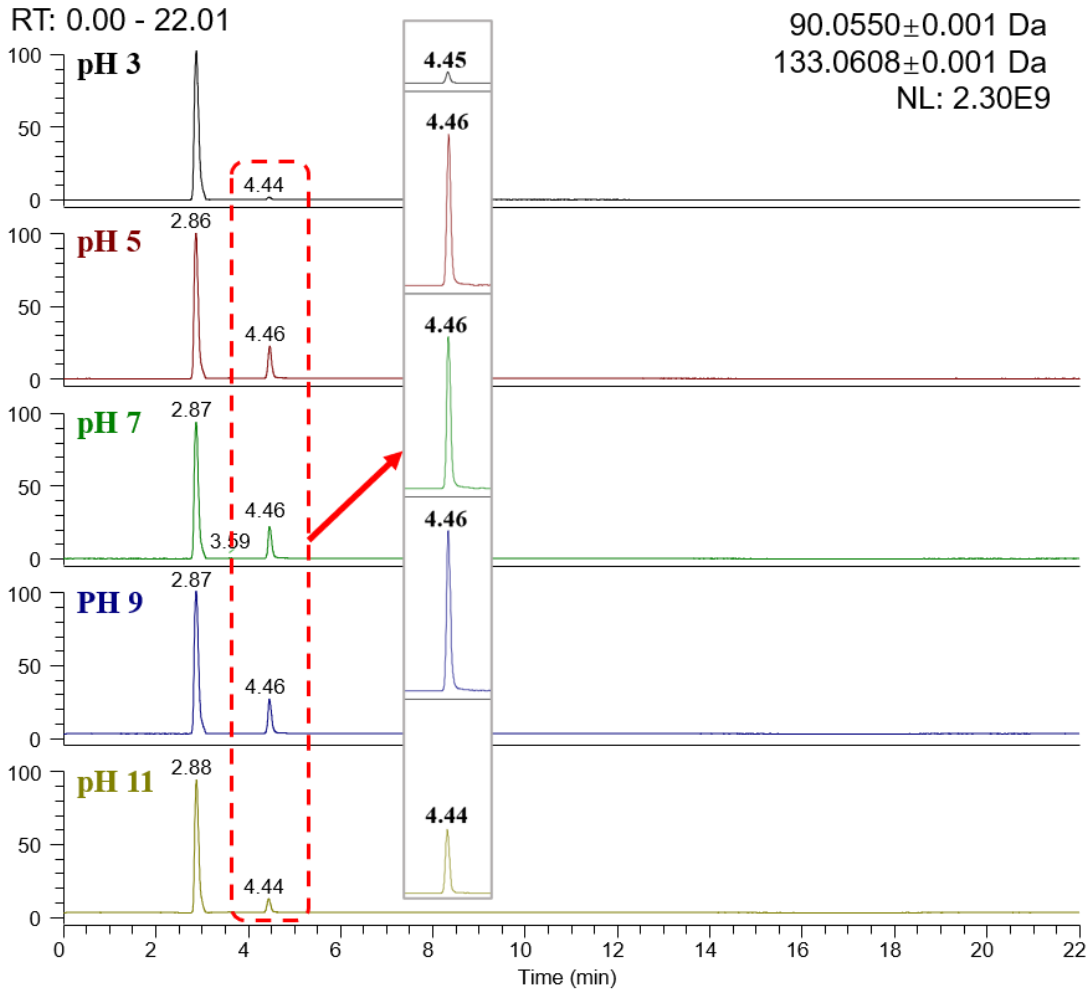

Figure S8. EIC-MS profile of Ala and carbamoyl-Ala under different pH conditions (3-11). HPLC conditions for single amino acid derivatives were same as Figure S5.

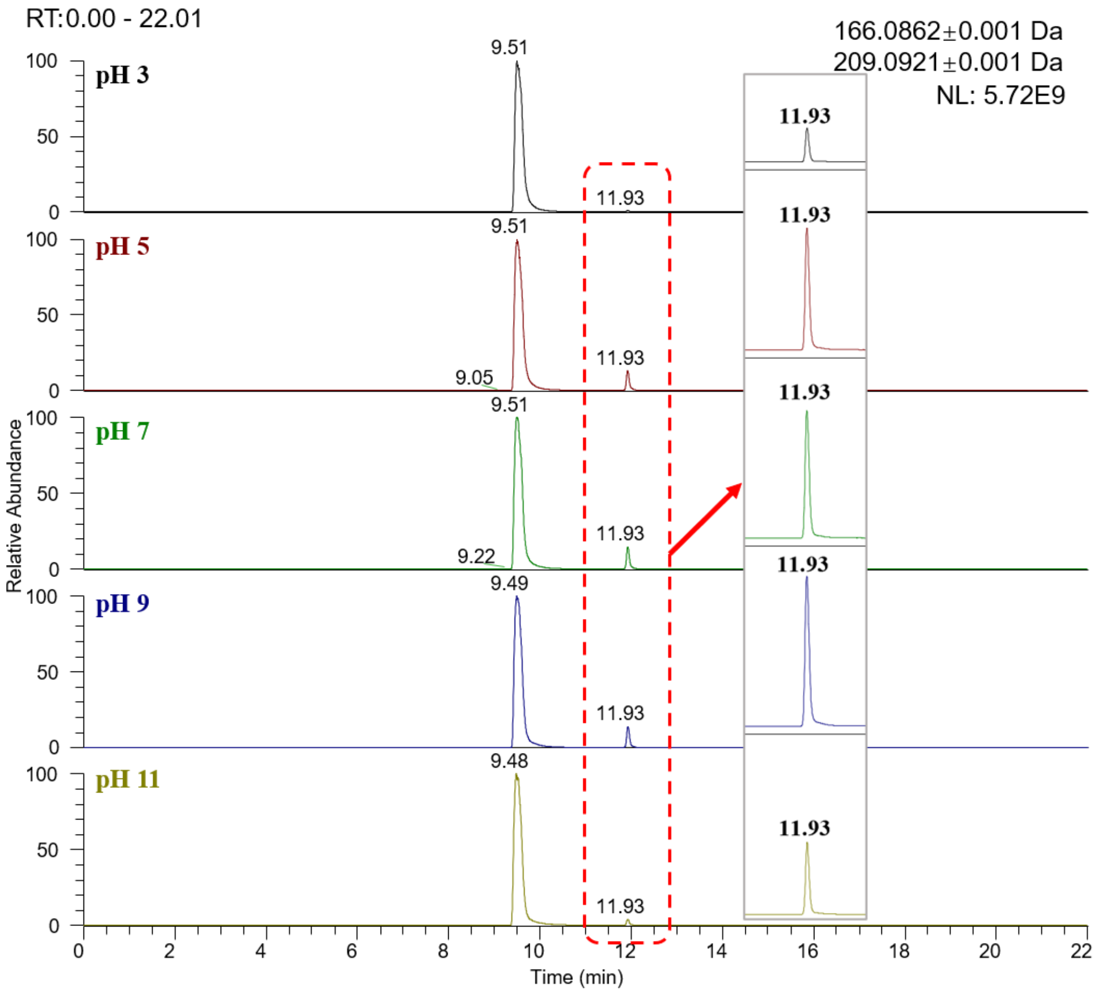

Figure S9. EIC-MS profile of Phe and carbamoyl-Phe under different pH conditions (3-11). HPLC conditions for single amino acid derivatives were same as Figure S5.

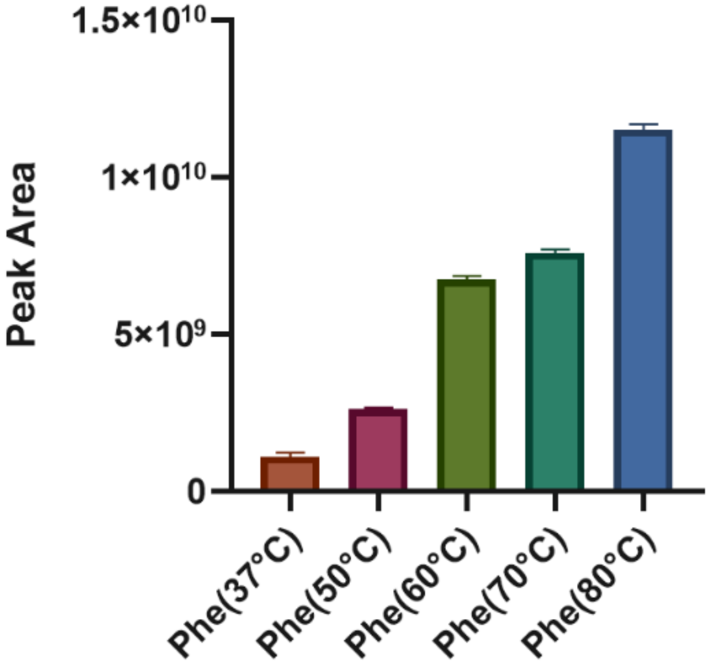

Figure S10. The yield of carbamoyl-Phe under different temperature conditions (37°C - 80°C).

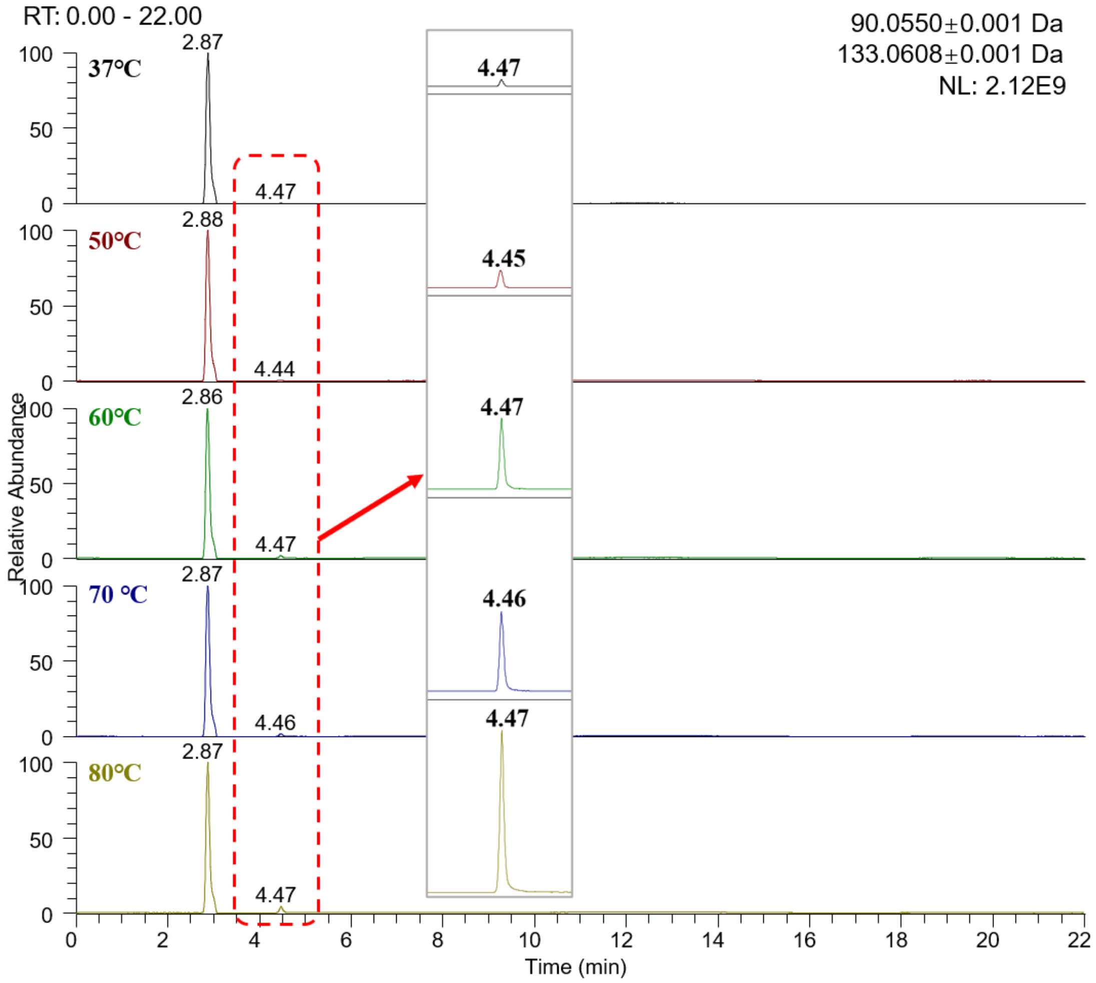

Figure S11. EIC-MS profile of Ala and carbamoyl-Ala under different temperature conditions (37°C -80°C). HPLC conditions for single amino acid derivatives were same as Figure S5.

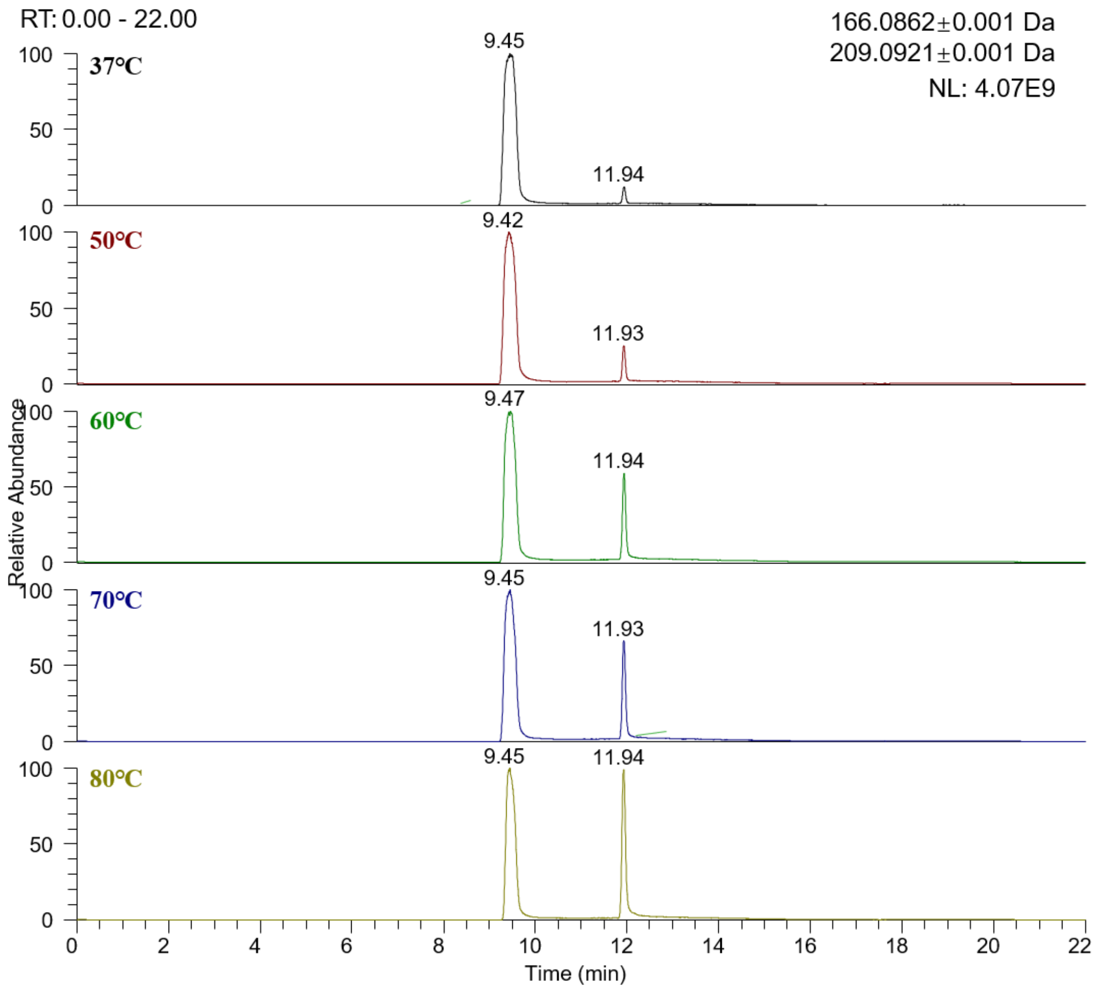

Figure S12. EIC-MS profile of Phe and carbamoyl-Phe under different temperature conditions (37°C -80°C). HPLC conditions for single amino acid derivatives were same as Figure S5.

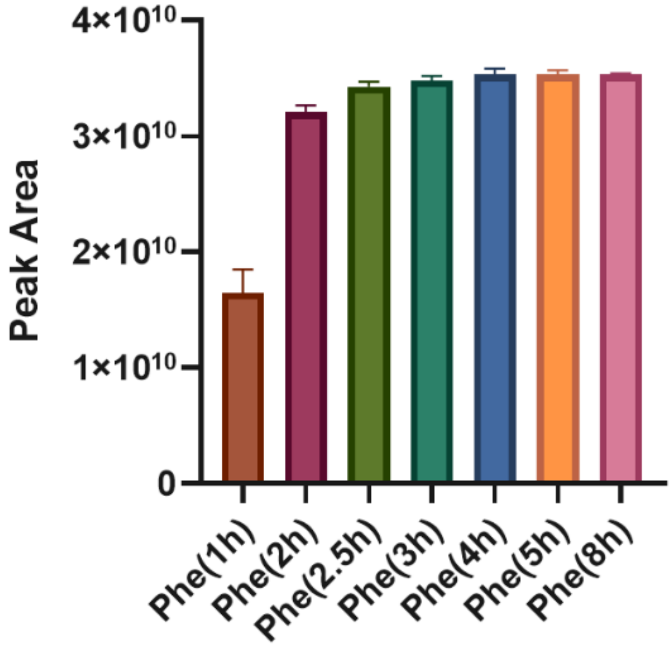

Figure S13. The yield of carbamoyl-Phe under different time conditions (1-8 h).

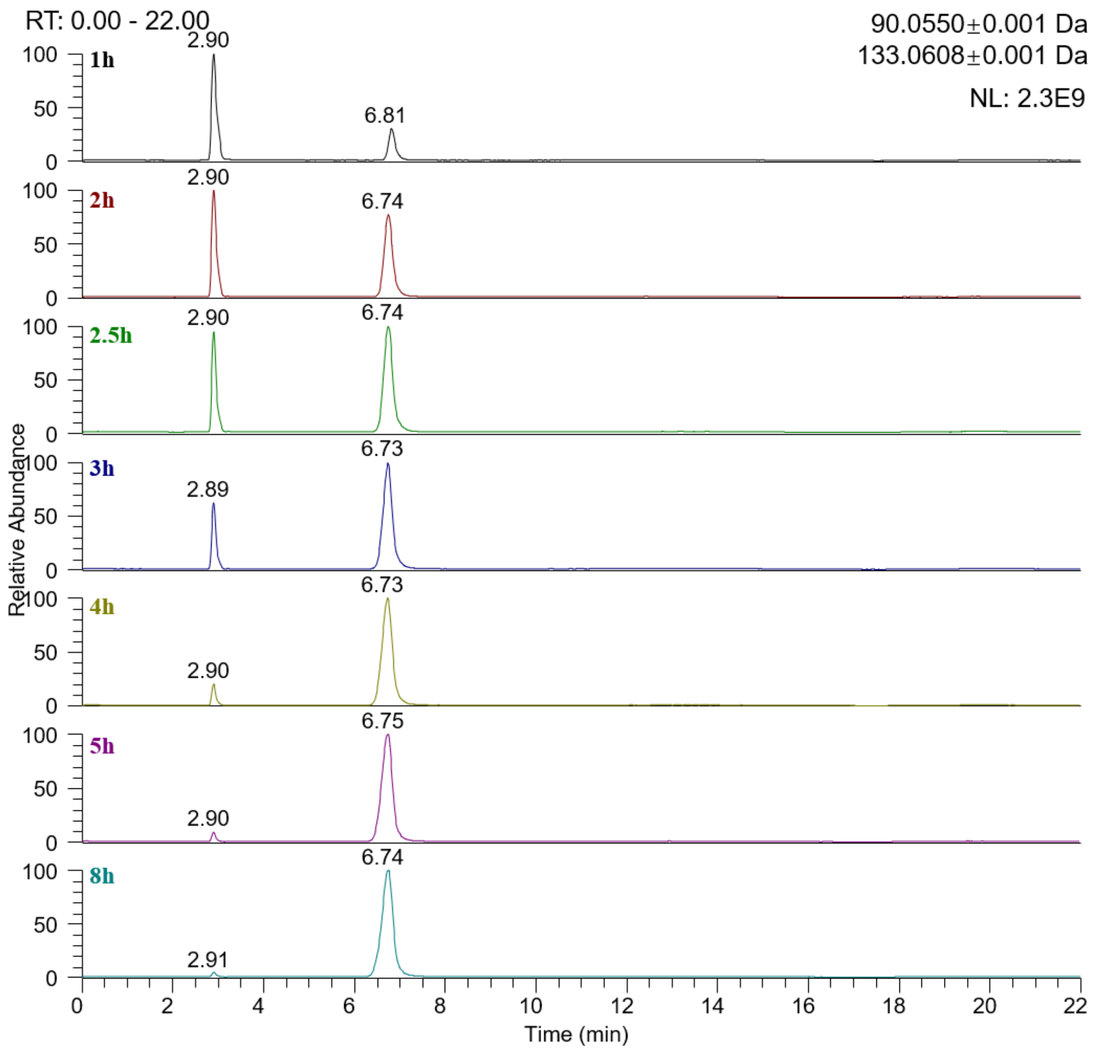

Figure S14. EIC-MS profile of Ala and carbamoyl-Ala under different time conditions (1-8 h). HPLC conditions for single amino acid derivatives were same as Figure S5.

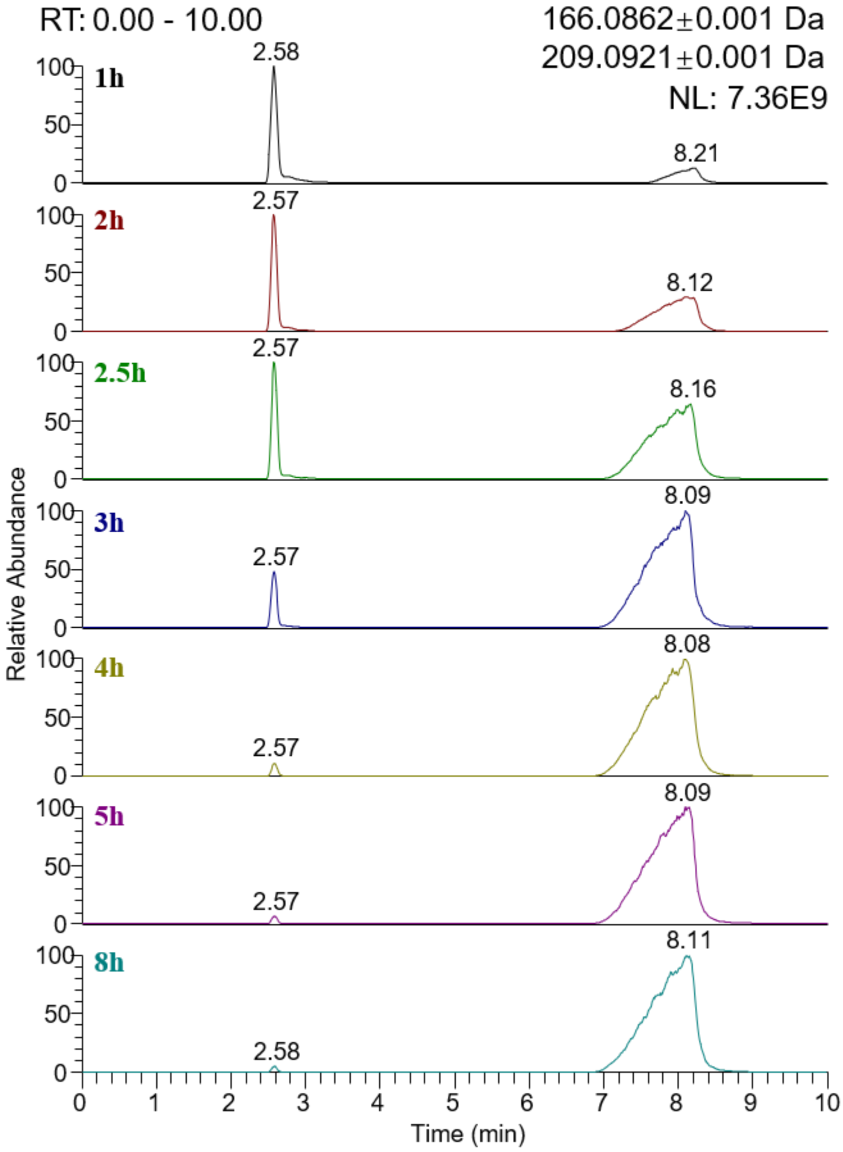

Figure S15. EIC-MS profile of Phe and carbamoyl-Phe under different time conditions (1-8 h). HPLC conditions for single amino acid derivatives was as follows: mobile phase A: deionized water (0.1% formic acid); mobile phase B: acetonitrile. Gradient program was as follows: 0-10 min, 50% B. The eluent flow rate was 1 mL /min and the column was maintained at 30°C and 2μL of the sample was injected.

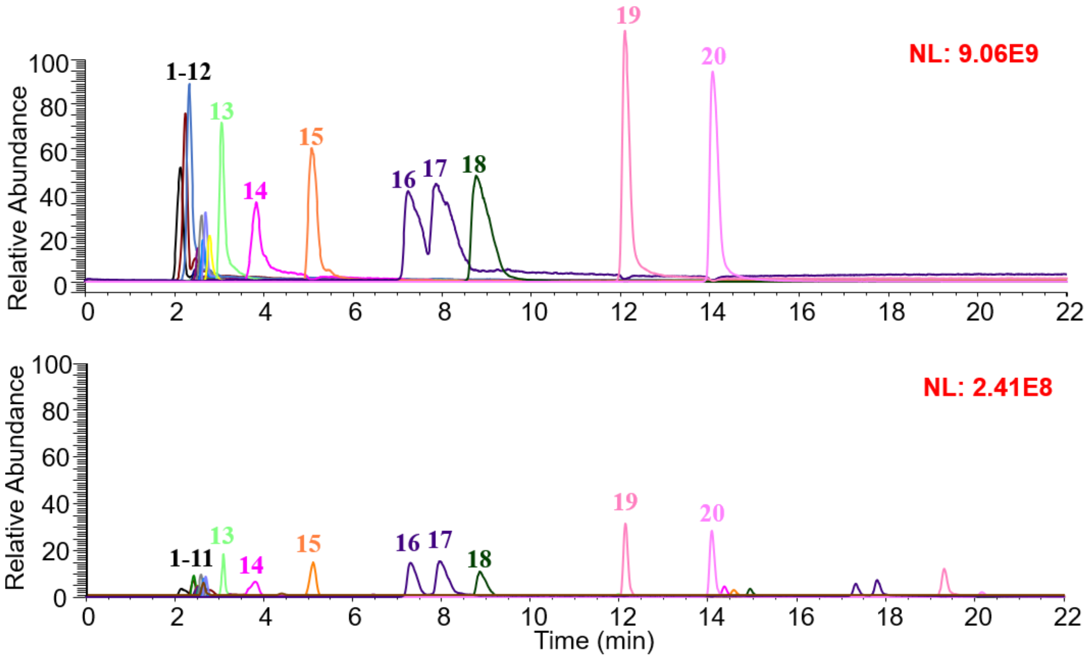

Figure S16. EIC-MS profile of 20 AAs before and after derivatization. a) EIC-MS profile of 20 AAs. b) EIC-MS profile of residual 20 AA in the derivate sample. AAs are marked with numbers as follows, 1: Lys, 2: His, 3: Arg, 4: Gly, 5: Ser, 6: Asn, 7: Ala, 8: Gln, 9: Asp, 10: Thr, 11: Glu, 12: Cys, 13: Pro, 14: Val, 15: Met, 16: Ile, 17: Leu, 18: Tyr, 19: Phe and 20: Trp. The m/z extraction range of AAs and CAAs were listed in Table S1.

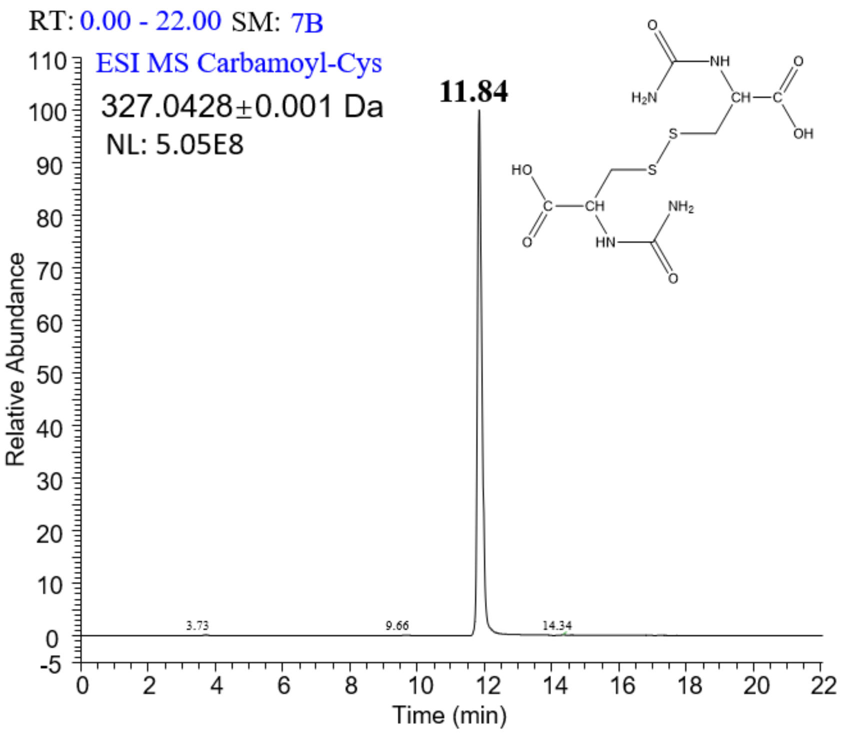

Figure S17. EIC-MS profile of 3,3'-disulfanediylbis (2-ureidopropanoic acid). HPLC conditions for derivatization of 20 kinds of amino acid mixed derivatives were as follows: mobile phase A: deionized water (0.1% formic acid); mobile phase B: acetonitrile. Gradient program was as follows: 0-5 min, 5%; 5-45 min, 5-90%; 45-50 min, 90-5%; 50-55min, 5% B. The eluent flow rate was 1 mL/min and the column was maintained at 30 ◦C and 8 μL of the sample was injected. 3,3'-disulfanediylbis(2-ureidopropanoic acid)

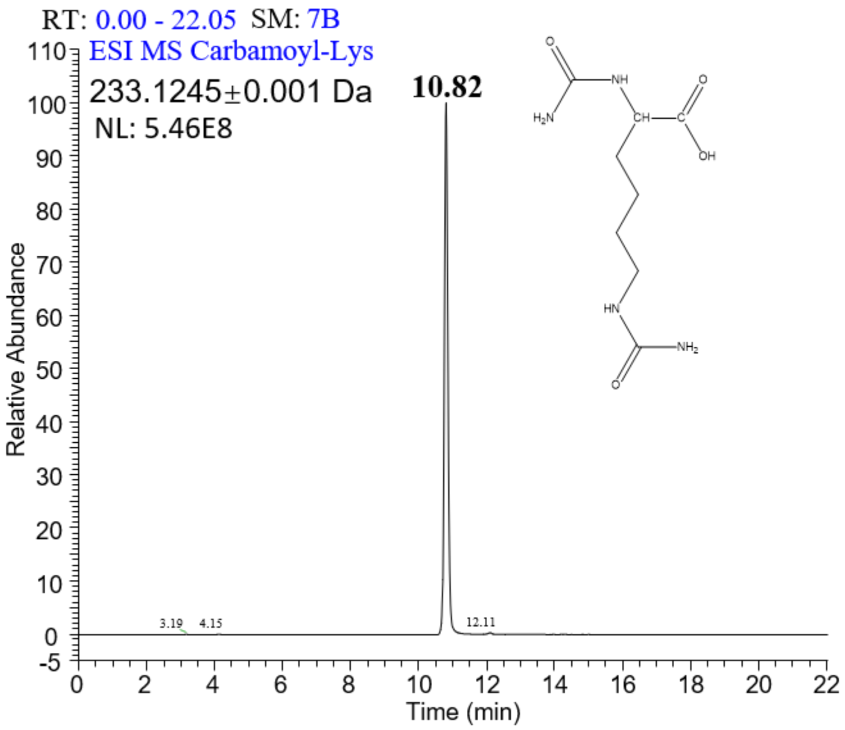

Figure S18. EIC-MS profile of N^2, N^6-dicarbamoyllysine. HPLC conditions for derivatization of 20 kinds of amino acid mixed derivatives were same as Figure S15.

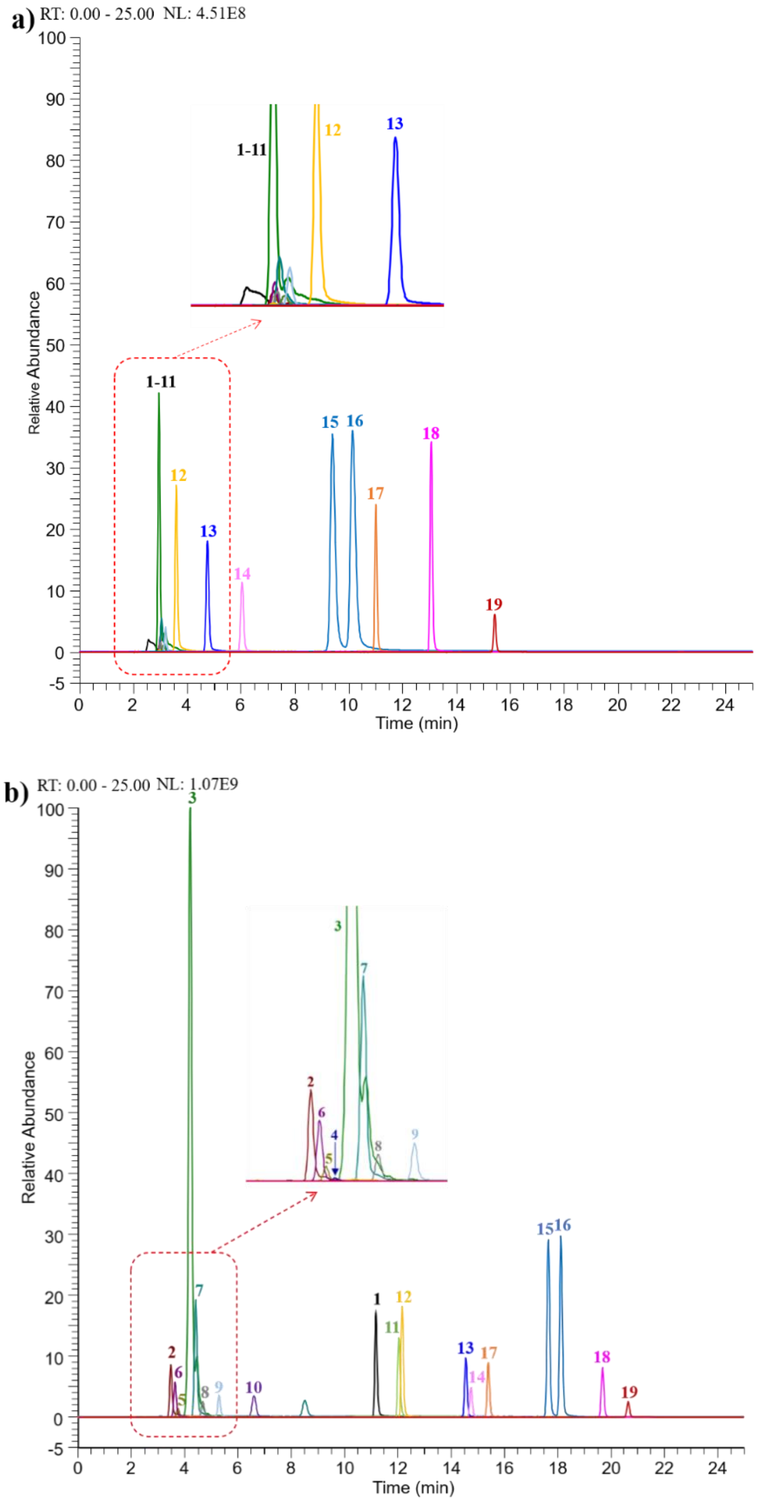

Figure S19. Derivatization of complex sample. a) EIC-MS profile of Cell culture medium RPMI 1640. b) EIC-MS profile of Cell culture medium RPMI 1640 after derivatization. Amino acids are marked with numbers as follows, 1: Lys, 2: His, 3: Arg, 4: Gly, 5: Ser, 6: Asn, 7: Gln, 8: Asp, 9: Thr, 10: Glu, 11: Cys, 12: Pro, 13: Val, 14: Met, 15: Ile, 16: Leu, 17: Tyr, 18: Phe and 19: Trp. The m/z extraction range of AAs and CAAs were listed in Table S1.

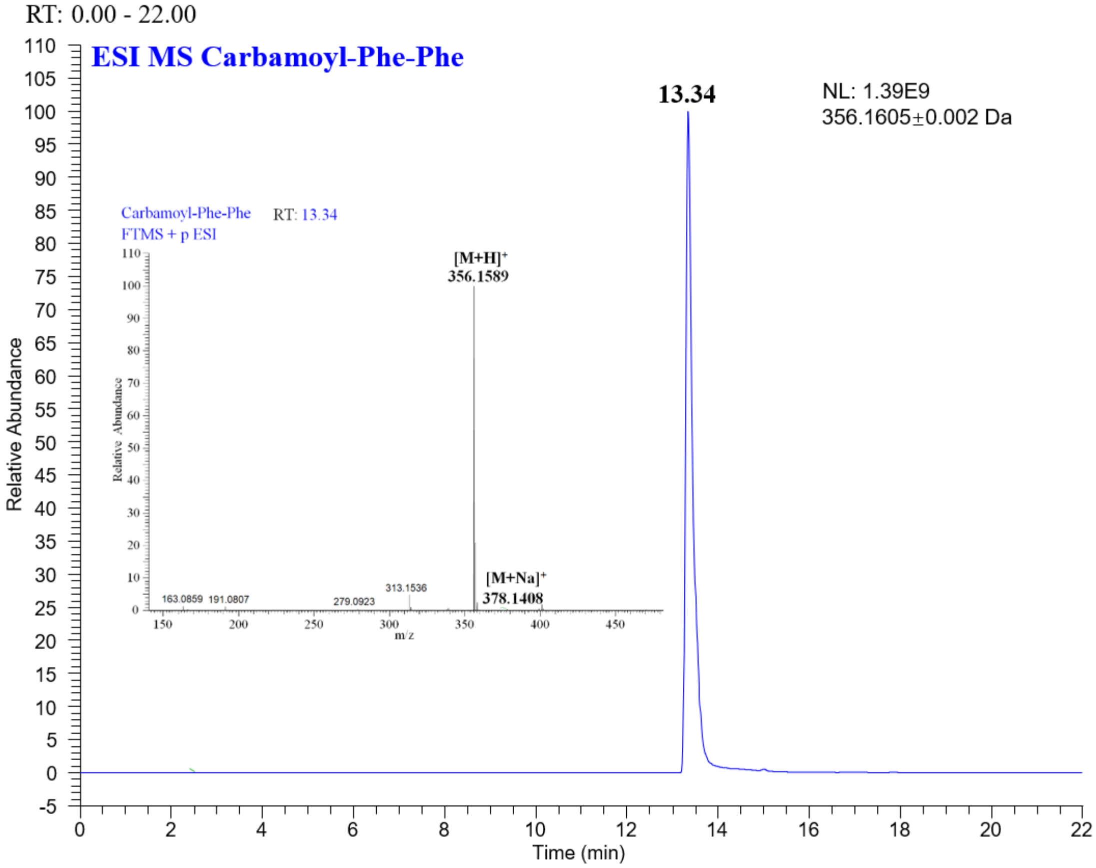

Figure S20. EIC-MS profile of carbamoyl-Phe-Phe. HPLC conditions for single amino acid derivatives was same as Figure S3.

Table S1 m/z extraction range of AAs and CAAs.

AA	[M+H]+[a]	[M+H]+[b]	extraction range [a]	extraction range [b]
Lys	147.1128	233.1245	147.1128±0.001 Da	233.1245±0.001 Da
His	156.0768	199.0826	156.0768±0.001 Da	199.0826±0.001 Da
Arg	175.1190	218.1248	175.1190±0.001 Da	218.1248±0.001 Da
Gly	76.0393	119.0451	76.0393±0.001 Da	119.0451±0.001 Da
Ser	106.0499	149.0557	106.0499±0.001 Da	149.0557±0.001 Da
Asn	133.0608	176.0666	133.0608±0.001 Da	176.0666±0.001 Da
Ala	90.0550	133.0608	90.0550±0.001 Da	133.0608±0.001 Da
Gln	147.0764	190.0822	147.0764±0.001 Da	190.0822±0.001 Da
Asp	134.0448	177.0506	134.0448±0.001 Da	177.0506±0.001 Da
Thr	120.0655	163.0713	120.0655±0.001 Da	163.0713±0.001 Da
Glu	148.0604	191.0662	148.0604±0.001 Da	191.0662±0.001 Da
Cys	122.0270	327.0428	122.0280±0.001 Da	327.0428±0.001 Da
Pro	116.0706	159.0764	116.0706±0.001 Da	159.0764±0.001 Da
Val	118.0863	161.0921	118.0863±0.001 Da	161.0921±0.001 Da
Met	150.0583	193.0641	150.0583±0.001 Da	193.0641±0.001 Da
Ile	132.1019	175.1077	132.1019±0.001 Da	175.1077±0.001 Da
Leu	132.1019	175.1077	132.1019±0.001 Da	175.1077±0.001 Da
Tyr	182.0812	225.087	182.0812±0.001 Da	225.087±0.001 Da
Phe	166.0862	209.0920	166.0862±0.001 Da	209.0920±0.001 Da
Trp	205.0972	248.1030	205.0972±0.001 Da	248.1030±0.001 Da

a: [M+H]+ and m/z extraction range of 20 AAs in Fig. 3, Fig. 4 and Fig. S19; b: [M+H]+ and m/z extraction range of 20 CAAs in Fig. 3, Fig. 4 and Fig. S19.

Table S2 Changes in retention time before and after AA derivatization

AA	retention time (a)	retention time (b)
Lys	2.18	10.82
His	2.27	2.78
Arg	2.33	2.96
Gly	2.48	3.59
Ser	2.54	3.55
Asn	2.55	3.25
Ala	2.58	3.27
Gln	2.61	4.03
Asp	2.61	4.43
Thr	2.66	5.02
Glu	2.70	6.21
Cys	2.80	11.84
Pro	3.06	11.82
Val	3.83	14.36
Met	5.09	14.69
Ile	7.24	17.33
Leu	7.89	17.80
Tyr	8.76	14.92
Phe	12.09	19.29
Trp	14.03	20.14

a: Retention times of 20 AAs on reversed-phase columns. b: Retention times of 20 CAAs on reversed-phase columns.

Table S3 Derivatized conversion rate of 20 AA Mixtures.

AA	Conversion rate (%)	AA	Conversion rate (%)
Lys	98.57%	Glu	99.37%
His	97.06%	Cys	~100%
Arg	98.68%	Pro	97.24%
Gly	98.27%	Val	98.30%
Ser	97.10%	Met	98.56%
Asn	99.59%	Ile	96.83%
Ala	99.10%	Leu	96.65%
Gln	97.64%	Tyr	99.09%
Asp	99.35%	Phe	96.46%
Thr	99.54%	Trp	96.30%

* The conversion rate of amino acid derivatization was obtained from the ratio of the residual peak area and the original peak area before and after the reaction. The residual peak area of Cys was not detected.

Table S4 Formulation of cell culture medium DMEM. Cat.No is 10-013 (CORNING). Type of cell culture medium is Liquid, 1x. The unit of components is mg/L.

**Components**			
**Amino Acids**			
L-Arginine • HCl	84.00	L-Methionine	30.00
L-Cystine•2HCl	62.57	L-Phenylalanine	66.00
L-Glutamine	584.00	L-Serine	42.00
Glycine	30.00	L-Threonine	95.20
L-Histidine • HCl • H_2O	42.00	L-Tyrosine • 2Na • $2H_2O$	103.79
L-Isoleucine	104.80	L-Tryptophan	16.00
L-Leucine	104.80	L-Valine	94.00
L-Lysine•HCl	146.20		
**Inorganic Salts**			
$CaCl_2$ (anhydrous)	200.00	NaCl	6400.00
KCl	400.00	NaH_2PO_4 • H_2O	125.00
$MgSO_4$ (anhydrous)	97.70	$NaHCO_3$	3700.00
Fe $(NO_3)_3$ • $9H_2O$	0.10		
**Vitamins**			
D-Calcium pantothenate	4.00	Pyridoxine•HCl	4.00
Choline chloride	4.00	Riboflavin	0.40
Folic acid	4.00	Thiamine•HCl	4.00
i-Inositol	7.20	Nicotinamide	4.00
**Other**			
D-Glucose	4500.00	Phenol red•Na	15
Sodium pyruvate	110.00		

Related information from www.corning.com/lifesciences/media.

Table S5 Formulation of cell culture medium RPMI 1640. Cat.No is 10-040 (CORNING). Type of cell culture medium is Liquid, 1x. The unit of components is mg/L.

**Components**			
**Amino Acids**			
L-Arginine	200.00	L-Lysine•HCl	40.00
L-Asparagine•H_2O	56.82	L-Methionine	15.00
L-Aspartic acid	20.00	L-Phenylalanine	15.00
L-Cystine•2HCl	65.20	L-Proline	20.00
L-Glutamic acid	20.00	L-Serine	30.00
L-Glutamine	300.00	L-Threonine	20.00
Glycine	10.00	L-Tryptophan	5.00
L-Histidine	15.00	L-Tyrosine•2Na•$2H_2O$	28.83
L-Isoleucine	50.00	L-Valine	20.00
L-Leucine	50.00	Hydroxy-L-proline	20.00
**Inorganic Salts**			
$Ca(NO_3)_2$•$4H_2O$	100.00	NaCl	6000.00
KCl	400.00	Na_2HPO_4 (anhydrous)	800.70
$MgSO_4$ (anhydrous)	48.80	$NaHCO_3$	2000.00
**Vitamins**			
Biotin	0.20	Pyridoxine•HCl	1.00
D-Calcium pantothenate	0.025	Para-Aminobenzoic acid	1.00
Choline chloride	3.00	Riboflavin	0.20
Folic acid	1.00	Thiamine•HCl	1.00
i-Inositol	35.00	Vitamin B_{12}	0.005
Nicotinamide	1.00		
**Other**			
D-Glucose	2000.00	Phenol red•Na	5
Glutathione (reduced)	1.00		

Related information from www.corning.com/lifesciences/media.

Table S6 Derivatized conversion rate of AAs in Cell culture medium DMEM.

AA	Conversion rate (%)	AA	Conversion rate (%)
Lys	98.63%	Val	98.55%
His	99.20%	Met	99.41%
Arg	99.15%	Ile	98.08%
Gly	~100%*	Leu	97.76%
Ser	99.23%	Tyr	99.33%
Gln	93.19%	Phe	99.46%
Thr	98.51%	Trp	99.33%
Cys	~100%*		

* The conversion rate of amino acid derivatization was obtained from the ratio of the residual peak area and the original peak area before and after the reaction. The residual peak area of Gly and Cys was not detected.
